# Substance P’s Impact on Chronic Pain and Psychiatric Conditions—A Narrative Review

**DOI:** 10.3390/ijms25115905

**Published:** 2024-05-28

**Authors:** Charles Humes, Aleksandar Sic, Nebojsa Nick Knezevic

**Affiliations:** 1 Department of Anesthesiology, Advocate Illinois Masonic Medical Center, Chicago, IL 60657, USA; charles.humes@my.rfums.org (C.H.); aca.smed01@gmail.com (A.S.); 2 Chicago Medical School, Rosalind Franklin University of Medicine and Science, North Chicago, IL 60064, USA; 3 Faculty of Medicine, University of Belgrade, 11000 Belgrade, Serbia; 4 Department of Anesthesiology, University of Illinois, Chicago, IL 60612, USA; 5 Department of Surgery, University of Illinois, Chicago, IL 60612, USA

**Keywords:** substance P, chronic pain, depression, anxiety, PTSD

## Abstract

Substance P (SP) plays a crucial role in pain modulation, with significant implications for major depressive disorder (MDD), anxiety disorders, and post-traumatic stress disorder (PTSD). Elevated SP levels are linked to heightened pain sensitivity and various psychiatric conditions, spurring interest in potential therapeutic interventions. In chronic pain, commonly associated with MDD and anxiety disorders, SP emerges as a key mediator in pain and emotional regulation. This review examines SP’s impact on pain perception and its contributions to MDD, anxiety disorders, and PTSD. The association of SP with increased pain sensitivity and chronic pain conditions underscores its importance in pain modulation. Additionally, SP influences the pathophysiology of MDD, anxiety disorders, and PTSD, highlighting its potential as a therapeutic target. Understanding SP’s diverse effects provides valuable insights into the mechanisms underlying these psychiatric disorders and their treatment. Further research is essential to explore SP modulation in psychiatric disorders and develop more effective treatment strategies.

## 1. Introduction

Pain is a distressing sensory and emotional experience associated with actual or potential tissue damage, or is described in terms that suggest such damage. Consequently, extensive research has focused on understanding the physiological and clinical processes of pain. This body of research has identified numerous neurotransmitters and chemical mediators, with Substance P being particularly notable. Substance P plays a critical role in the development, modulation, and progression of both acute and chronic pain. Studies have demonstrated the effects of pain on various physiological processes, including those affecting the mind [1]. Given the widespread physiological impact of pain, questions have arisen regarding whether neurotransmitters involved in pain also influence disease processes linked to pain, such as psychiatric conditions. This manuscript aims to address these questions by exploring the molecular and neurobiological pathways of Substance P and its effects on the body’s physiological processes. It will further examine studies that elucidate the interplay between Substance P and psychiatric conditions, including depression, anxiety, and post-traumatic stress disorder (PTSD). Additionally, the manuscript will review research on how targeting Substance P and its pathways might help treat these disease processes. By providing a comprehensive overview of Substance P’s role in both pain and psychiatry, this manuscript aims to contribute to the existing literature and highlight areas requiring further research. Ultimately, we hope to advance the understanding of Substance P’s vast implications and support the development of prevention and treatment strategies for the conditions discussed.

## 2. Substance P’s History and Molecular Pathway

### 2.1. History

Substance P was first discovered in 1931 by Von Euler and Gaddum, who isolated it from the equine brain and gut. In the 1970s, it was also isolated from hypothalamic bovine tissue [1]. At that time, Substance P was identified as a neuropeptide, specifically a tachykinin. Two other tachykinins, neurokinin A and neurokinin B, were identified shortly thereafter [1]. Tachykinins are found ubiquitously in mammalian body tissues, with Substance P particularly concentrated in the dorsal horn of the spinal cord, the substantia nigra, and the amygdala [1]. The name “tachykinins” derives from the prefix “tachy”, meaning swift, reflecting these neuropeptides’ ability to evoke rapid responses in neurons [2]. 

### 2.2. Synthesis and Release

Tachykinins are synthesized through the alternate processing of two *TAC* genes: the *preprotachykinin I* (*PPTI)* gene, which synthesizes both Substance P and neurokinin A, and the *preprotachykinin II* (*PPTII)* gene, which synthesizes neurokinin B [2]. Activation of these genes leads to alternative RNA splicing for *PPTI*, resulting in four different mRNA forms: alpha, beta, gamma, and delta [3]. These forms are precursors to Substance P, while neurokinin A and B can only be synthesized from the gamma and beta forms [3]. The spliced mRNA then undergoes translation and cleavage to form the peptide [3]. 

This synthesis occurs in small and medium-sized neurons [2]. After synthesis, the neuropeptides are stored in dense core vesicles and transported to central and peripheral nerve terminals via fast axonal transport [2]. Signals such as inflammation or injury trigger the release of these peptides into the synaptic cleft, where they bind to their respective receptors and propagate tachykinin effects [2]. 

### 2.3. Tachykinin Receptors

After synthesis, storage, and release, tachykinins bind to three distinct receptors, labeled NK1, NK2, and NK3 [3]. Substance P shows the highest potency when binding to the NK1 receptor, while neurokinin A and neurokinin B preferentially bind to NK2 and NK2, respectively [4]. Despite their preferential binding, all three tachykinins can interact with any of the three receptors due to the conformational flexibility of their short, linear peptide structures [5]. Additionally, these peptides share a conserved carboxy-terminal sequence essential for neurokinin receptor activation, enabling them to interact with the NK1 receptor. 

Anatomically, NK1 receptors are widely expressed across various brain regions, including the raphe nucleus, hippocampus, locus coeruleus, nucleus accumbens, nucleus reticularis paragigantocellularis, and striatum [6,7]. In contrast, NK2 and NK3 receptors are less prevalent within the central nervous system (CNS) [6,7]. Substance P operates through volume transmission, meaning it does not need to be in close proximity to its receptors and can be transported to distant targets, facilitating a broader anatomical distribution [8]. The interaction between tachykinins and their neurokinin receptors results in high-affinity binding, triggering a cascade of metabolic events.

### 2.4. Metabolic Cascade of Substance P

**Figure 1:**
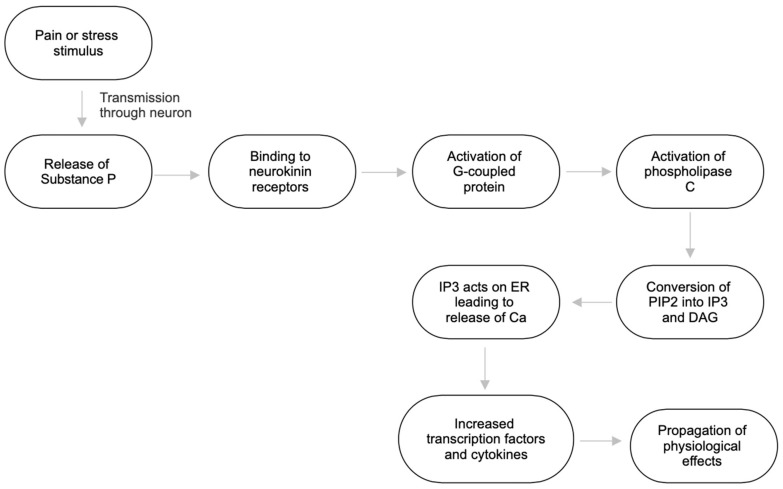
Molecular pathway of Substance P with effects [1,2,3,4,5,6,7,8,9,10,11].

Neurokinin receptors, including NK1, belong to the seven-transmembrane G protein-coupled receptor [9]. These receptors consist of seven hydrophobic transmembrane domains, three extracellular domains, and three intracellular loops, with the N-terminus tail positioned extracellularly and the C-terminus tail intracellularly [9]. This manuscript focuses particularly on NK1 due to its high affinity for Substance P [9]. When substance P binds to the Gq-coupled NK1 receptor, it activates the G protein, which subsequently activates phospholipase-CB (PLCB) [9]. Phospholipase-CB in turn converts phosphatidylinositol 4,5-bisphosphate (PIP2) into inositol 1,4,5 triphosphate (IP3) and diacyl-glycerol (DAG) [6]. IP3 acts on the endoplasmic reticulum, causing calcium release into the cytosol [9]. This increase in intracellular calcium leads to enhanced gene transcription and affects various physiological processes [4]. Activation of this cascade also upregulates cytokines and transcription factors, such as nuclear factor kappa-light-chain-enhancer of activated B cells (NF-kB) [10]. These molecules increase pro-inflammatory factors, contributing to pain development [10]. The chain reaction initiated by Substance P and other tachykinins propagates throughout the cells, potentiating their effects and influencing the physiological processes discussed in this manuscript (Figure 1). 

### 2.5. Anatomic Localization of Metabolic Effects

Substance P is prevalent throughout the CNS, with high concentrations in areas such as the nucleus raphe, nucleus reticularis paragigantocellularis, hypothalamus, and nucleus accumbens [6,7,8]. It is also present in the peripheral nervous system, particularly in the dendritic spines of free nerve endings, playing a crucial role in the pain processing pathway [1]. The localization of neurokinin receptors, especially NK1, significantly influences the effects mediated by Substance P. Moreover, the interaction between Substance P and other neurotransmitters, such as dopamine in the substantia nigra and nucleus accumbens, and serotonin in the locus coeruleus, further potentiates the effects of Substance P [11]. This manuscript will further explore the interplay between these neurotransmitters and Substance P in both the central and peripheral nervous systems.

## 3. Animal Models

Animal models have been crucial in understanding Substance P and its roles in various physiological processes. This research approach allows scientists to stimulate and observe the effects of Substance P and pain, offering valuable insights into these mechanisms [12,13]. 

One significant animal model examined chronic pain and the upregulation of neurokinin receptors, specifically NK1. In this experiment, mice were subjected to water stress and sham stress for one hour daily over ten consecutive days [12]. Simultaneously, they received injections of markers to identify the upregulation of key factors in the pain process [12]. The results demonstrated an upregulation of the NK1 receptor in the spinal cord, attributed to increased activity of spinal microglia [12]. The researchers concluded that the upregulation of NK1 receptors, along with increased Substance P, played a crucial role in the development of hyperalgesia in the context of chronic stress.

Another animal model explored the effects of Substance P on anxiety and depression, a topic further examined in this manuscript. This model focused on the diverse effects of Substance P following microinjections into various brain regions [13]. The study found that Substance P administration produced an anxiogenic effect in mice. Particularly when injected into the periaqueductal gray [13]. Conversely, applying NK1 antagonists to these mice resulted in an anxiolytic effect [13]. Furthermore, studies showed that mice lacking the gene for Substance P production were less prone to developing symptoms of depression and anxiety [14].

These animal models, summarized in Table 1, enhance our understanding of Substance P’s roles in the body and its impact on individuals. They highlight the wide range of effects induced by Substance P and suggest avenues for further research to identify prevention and treatment strategies for conditions associated with Substance P.

## 4. Understanding Substance P: Molecular Mechanisms and Physiological Pathways

Much has been discussed regarding the molecular mechanisms and pathways by which Substance P exerts its effects. Understanding these pathways is critical to comprehending how Substance P functions on a physiological level. Although the role of Substance P in cortical, subcortical, and descending pathways is still being investigated, current findings are crucial for understanding its function and clinical implications.

### 4.1. Pain Transmission and Substance P

**Figure 2:**
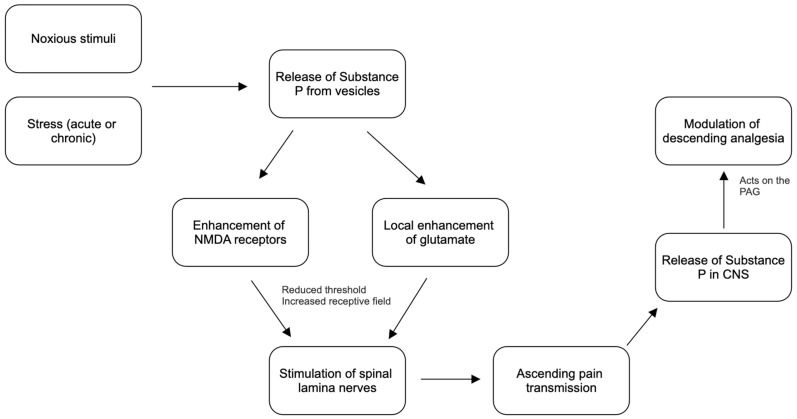
Mechanism for Substance P’s effect on pain transmission in both ascending and descending pathways [1,17,18,19,20,21,22,23,24,25,26,27,28,29,30,31].

The process of pain begins with a stimulus sensed by primary afferent nociceptors, the free nerve endings present in body tissues [17]. This stimulus is transmitted along fast, myelinated, and slow, unmyelinated axons into the spinal cord, where it synapses in the dorsal horn [17]. This synapse involves neurotransmitters, particularly Substance P and glutamate [17,18]. Substance P, localized in vesicles within dendritic shafts and spines, projects to make axodendritic connections between neurons [18]. The propagation of the stimulus to these dendritic spines triggers the release of Substance P, which can act locally or by volume transmission on neurons throughout the body [18]. Research indicates that Substance P targets spinal lamina neurons 1 and 5, which project to multiple brain sites, including the thalamus and periaqueductal gray [19]. Moreover, Substance P induces an increase in NMDA-activated depolarization of neurons, vital for developing algesia [1,20]. Substance P binds to neurokinin receptors, initiating a cascade of molecular intermediaries that affect neurons and other neurotransmitters. Studies on Substance P antagonists have shown decreased pain levels and modulation, supporting the role of Substance P as both an excitatory neurotransmitter and a pain modulator [1,21]. Furthermore, there are other manners in which Substance P can affect the physiological pathways of pain (Figure 2). 

### 4.2. Substance P and Glutamate Enhancement

Substance P also modulates pain by enhancing glutamate’s effects [1,22]. This enhancement results from increased phosphorylation of NMDA receptors due to neurokinin 1 receptor activation of phospholipase C [20]. Additionally, Substance P increases glutamate-induced currents in major neurons [1,22]. Glutamate, the main excitatory neurotransmitter in the spinal cord, contributes to nociceptive stimulus transmission [23,24]. The nociceptive stimulus then ascends the spinal cord in the spinothalamic tract before synapsing in the thalamus and the medial reticular formation of the brainstem [25,26]. These ascending tracts synapse within the thalamus’s ventrocaudal and medial aspects, with the medial aspect also receiving input from the spinoreticular pathway. Following the thalamus, the pain stimulus is projected to the primary somatosensory cortex, where it is registered and analyzed [25,26]. 

### 4.3. Descending Pathways and Substance P

The descending pathway is critical for somatosensory transmission back to the dorsal horn as well as for pain modulation [27]. The descending pathway projects from the somatosensory cortex to the dorsal horn via the rostral ventromedial medulla. Most importantly, the rostral ventromedial medulla contains the periaqueductal gray (PAG) [27]. The PAG is a site that plays a major role in the actions of opioids and cannabinoids and their effects in modulating the pain pathways [27]. As previously mentioned, NK1 receptors are heavily localized to the PAG, making it an ideal spot for Substance P to act. Studies have shown that the application of Substance P appears to produce potent analgesia when injected directly into the PAG [16]. Furthermore, other studies have found that when exposed to noxious stimuli and behavioral stress, endogenous Substance P is released [28]. Substance P interacts with NK1 receptors in the body to help mediate descending analgesia from the PAG [28]. This mediation of descending analgesia is thought to be secondary to the enhanced action potential frequency of PAG neurons [28]. The enhanced action potential frequency results from a reduced inward potassium conductance [28]. Furthermore, additional evidence for this effect of Substance P was seen with the use of functional magnetic resonance imaging (fMRI) [1]. When stress and pain were applied, fMRI demonstrated increased activation of Substance P-related descending pathways [1]. These theories on Substance P’s role in the PAG were lent credence when it was demonstrated that NK1 receptor antagonists lead to decreased levels of analgesia [16,28]. Furthermore, these studies demonstrated that Substance P suppressed GABAergic activity in the PAG by activating local glutamate circuits and endocannabinoid signaling leading to analgesia when stimulated by noxious stimuli or stress [16].

A third and equally important manner in which Substance P affects the body’s perception of pain is located in the periphery. As discussed prior, noxious stimuli or stress lead to the release of Substance P and glutamate from vesicles [18]. Furthermore, Substance P also enhances the effects of glutamate’s actions within the synapse [18]. A secondary action of both neurotransmitters is their ability to lower the pain threshold for the dorsal horn neurons they interact with [1,29]. This allows the dorsal horn neurons to be stimulated more easily by the stimuli or their stress if it continues [1,29]. Recent studies have strived to confirm that Substance P reduces the threshold of neurons to experience pain. A study was conducted on rats where electroacupuncture was applied as a means to increase the pain threshold [30]. Results demonstrated that this application could reduce the levels of Substance P and NK-1 receptors, contributing to a raised pain threshold [30]. Furthermore, the study showed that microinjections of Substance P antagonists produced similar responses as the electroacupuncture, further solidifying the theory that Substance P plays a role in reducing the pain threshold [30]. Moreover, this reduced threshold is further implicated in the plasticity of the neurons themselves, increasing the outputs of the neurons and contributing to the development of chronic pain [1,31]. Concurrently, as Substance P works to decrease the pain threshold of these neurons, it is also working on increasing their receptive fields [31]. Receptive fields are specific regions of sensory space in which a stimulus can elicit a response from the sensory neuron [31]. Increased receptive fields are associated with a wider range of the body in which the individual can detect the noxious stimulus [31]. The wider range of the receptive field leads to further excitability of the neurons and transmission of pain [31]. Again, recent research demonstrates that this increased receptive field and reduced threshold results from the Substance P-mediated phosphorylation of G proteins and subsequent production of chemical mediators [20]. 

There are some limitations to these theories of Substance P’s effects on sensitization, enhancing glutamate, and increasing neuronal receptive fields. One particular limitation of note is that while studies have been able to determine the effects of Substance P, the exact mechanism by which these effects occur is unknown [1,18,29]. As stated previously, studies and the literature have demonstrated that Substance P works as a G-coupled receptor in a metabolic system; it is unclear what intermediates help contribute to the effects seen on neurons in the peripheral nervous system [1,18,29]. This lack of knowledge and understanding presents an opportunity for further research to investigate these mechanisms more clearly. Given the wide range of effects that Substance P has on a neuronal level, research that illuminates the precise mechanisms of Substance P’s effects could provide avenues for the prevention and treatment of pain and other disease processes associated with Substance P. Another limitation in these studies is not being able to distinguish which aspect or type of neuron is most affected by Substance P, be it an interneuron or laminae neuron [1,18,29]. This provides opportunities for further research concerning the proportionality of the effect of Substance P on different neurons in the periphery. This information would provide more selective targets for possible prevention and treatment of Substance P-associated disease processes.

## 5. Acute, Chronic, and Neuropathic Pain

Chronic pain presents a significant health challenge, often accompanied by a high prevalence of associated conditions, such as anxiety, depression, insomnia, and cognitive dysfunction. This combination often amplifies the overall burden on individuals’ well-being [32]. It also increases disruptions in sleep patterns [33]. Chronic pain currently affects over 30% of individuals globally and is recognized as a distinct disease with significant personal and economic implications [34]. It also poses a substantial concern among the elderly population, with over 50% affected and up to 80% of nursing home residents impacted [35]. Moreover, chronic pain constitutes a frequent reason for seeking medical care, accounting for a substantial portion of general practitioner consultations, ranging from 22% to 50% [36]. Pain classification, though beneficial, often encounters overlaps due to this complexity. This complexity emphasizes the importance of the biopsychosocial model, which underscores the interaction of biological, psychological, and social factors in pain perception [34]. In recent research, the bidirectional relationship between chronic pain and psychological distress has been highlighted. The research underscores the significance of resilience factors, such as social support and good health, in mitigating the effects of chronic pain [37]. Recognizing the bidirectional relationship between pain and psychological distress is paramount, with resilience factors such as social support and good health mitigating its effects [35]. Recent findings indicate that survivors of critical illness, including COVID-19 patients, often experience chronic pain, with prevalence rates varying widely [38]. Challenges in pain management persist in intensive care unit (ICU) settings and have been exacerbated by the demands of the pandemic. Factors such as prolonged immobilization and neuromuscular weakness contribute to the risk of chronic pain development, highlighting the necessity of early, multidisciplinary intervention [38]. Also, in addressing the management of chronic pain, studies have been conducted to evaluate the efficacy and potential harm of opioid treatments compared to placebo, non-opioid pharmacological therapies, and of various opioid dosing methods [39]. The findings indicated that while opioids offered modest benefits in short-term pain relief, function improvement, and sleep-quality enhancement compared to a placebo, they also carried heightened risks of adverse events, such as gastrointestinal issues, somnolence, and dizziness [39]. Observational studies emphasized the association between opioids and serious risks, including abuse, dependence, overdose, and mortality [39]. The research revealed that opioid therapy did not exhibit superiority over non-opioid medications in short-term outcomes, suggesting a need for further exploration of alternative treatments [39]. The study showed the critical importance of developing precise risk prediction tools, effective risk mitigation strategies, and optimal opioid tapering methods to address the substantial risks associated with long-term opioid therapy [39].

Among frequent comorbidities, chronic pain and mood disorders, particularly major depressive disorder (MDD), significantly diminish individuals’ overall well-being and quality of life [40]. Despite their substantial contribution to global disability, the precise mechanisms underlying their coexistence are still unknown [41]. Recent advancements in biochemical, cellular, and imaging technologies have revealed shared pathophysiological mechanisms between chronic pain and depression [42]. Kuner and Flor (2016) highlight that chronic pain is associated with long-term changes in neuronal circuits within the central nervous system (CNS), leading to alterations in perception and behavior. This suggests that chronic pain involves not only physical injury but also complex neuroplastic changes. Additionally, the role of synaptic plasticity, particularly long-term potentiation (LTP) and long-term depression (LTD), in modulating associative memory related to pain experiences underscores the interplay between pain and cognitive processes [1]. Moreover, the influence of neurotransmitters like glutamate and neuropeptides such as Substance P (SP) in enhancing LTP further elucidates the molecular mechanisms underlying pain sensitization and synaptic plasticity [1]. Research suggests that depression in chronic pain patients may differ from depression in those without pain [43]. Depressed chronic pain patients exhibit a recall bias for illness-related stimuli, while non-pain depressed patients show a bias for depression-related cues. Additionally, psychological responses such as catastrophizing and thought suppression play a role in linking chronic pain to depression [44]. Furthermore, chronic pain in adults in the USA is associated with higher severity scores for anxiety and depression, as measured by validated surveys in a nationally representative sample [44]. In addition, chronic pain is linked to adults taking medication for depression and/or anxiety [44].

Existing research suggests that Substance P (SP) may play a significant role in the development and progression of major depressive disorder (MDD), hinting at potential novel treatment avenues [45]. Additionally, a brief summary of relevant NK antagonist clinical trials is provided in Table 2. Multiple studies have noted elevated levels of SP in both serum and cerebrospinal fluid samples from individuals diagnosed with MDD. Prolonged antidepressant use has been associated with reduced SP synthesis in specific brain regions [46,47]. 

On the other hand, neuropathic pain commonly arises from damage or disease affecting the central or peripheral somatosensory nervous system. Peripheral neuropathic pain is especially prevalent and often associated with conditions like diabetic neuropathy, [48]. It represents a highly debilitating condition, impacting approximately 7% of the general population [49]. In the last 15 years, neuropathic pain research has been vibrant and productive, with researchers exploring various aspects of these complex and enduring pain conditions [50]. Microglia, vital for regulating functions in the central nervous system (CNS), play significant roles in both physiological and pathological contexts. Notably, in neuropathic pain, microglia emerge as key players, influencing its development and maintenance [51]. Furthermore, specific non-pharmacological strategies, notably neuromodulation, may provide relief, particularly in cases where conventional treatments have been ineffective. Recent research indicates that adjusting primary sensory input could represent a significant step forward in enhancing the management of central neuropathic pain. It is probable that, for the majority of patients, successful treatment will involve a combination of therapeutic approaches [52,53].

Contrary to chronic pain, which persists for a minimum of three months, acute pain typically stems from sudden trauma, acute medical conditions, or treatment. The reciprocal relationship between mood disorders and acute pain has garnered increasing attention, as both influence each other as risk factors. Depression and anxiety are linked to heightened perceptions of pain severity, while prolonged acute pain exacerbates mood dysregulation. Although depression and anxiety are both associated with acute pain, the connection with depression has received more extensive research. Pain often goes unrecognized as the primary complaint in depressed patients seeking primary care. However, studies on experimentally induced pain perception in depressed individuals present conflicting findings, showing both increased and decreased pain thresholds and tolerances. While less data are available on anxiety and pain, consistent evidence indicates that heightened anxiety is associated with increased pain severity and decreased pain tolerance. Anxiety, fear, stress, and catastrophizing are acknowledged as influential factors that mediate the connection between pain and disability [54].

## 6. Major Depressive Disorder

Major depressive disorder (MDD) is prevalent in the Western world, affecting around 12% of individuals [55]. This number is expected to rise with emerging data from the post-COVID-19 era [56]. Additionally, MDD is closely linked with acute and chronic pain conditions [44]. Consequently, research has begun exploring the role of Substance P in MDD. Diagnosis of MDD typically relies on clinical criteria, encompassing symptoms such as changes in sleep, appetite, loss of interest in activities, and suicidal ideation, among others [57]. These symptoms often cause significant functional impairment [56]. Psychiatric conditions like depression may not fit neatly into discrete categories but rather exist along a spectrum, ranging from mild to severe manifestations [58]. This spectrum challenges the traditional classification of disorders and encourages a more dimensional approach to understanding psychiatric illness. By viewing affective disorders through this lens, clinicians may gain a richer understanding of the complexity and heterogeneity of these conditions, potentially leading to more effective management strategies. On the other hand, the absence of a single reliable biomarker for psychiatric diagnoses, despite decades of research efforts, underscores the ongoing uncertainties surrounding the deep processes of the human mind and brain, as well as the complex nature of psychiatric disorders [58]. 

MDD involves an imbalance of neurotransmitters in the central nervous system (CNS), particularly serotonin, norepinephrine, and dopamine [59]. Treatment aims to increase these neurotransmitters by inhibiting their reuptake at the molecular level [59]. Major depressive disorder develops at a higher rate in patients who suffer from stress or pain. Studies have found that between 30 and 45% of individuals who met the criteria for MDD also had a chronic pain condition [59]. This literature review, confined to PubMed, may overlook relevant studies from other databases or unpublished sources, suggesting a potential publication bias. The search terms used might not capture all pertinent research, and unclear inclusion criteria could limit comprehensiveness. Interpretation bias may also influence how findings are synthesized and presented [60]. 

Recent efforts have identified Substance P as a potential contributor to MDD pathology. Substance P levels are elevated in individuals with MDD, irrespective of concurrent pain conditions [47]. In this study, 23 individuals with major depression and 33 controls had their Substance P (SP) levels measured using an enzyme immunoassay. Depressed participants’ levels were assessed at three intervals: before treatment, at 2 weeks, and at 4 weeks into therapy, while controls were measured at the start and after 4 weeks. In patients with major depressive disorder, regardless of a concurrent pain condition, increased levels of Substance P in the serum and the cerebrospinal fluid were discovered [47]. Moreover, this increased level was significantly higher than the control subjects who did not have major depressive disorder [47]. However, the study’s small sample size and lack of diversity may limit generalization. Focusing on short-term changes with treatment might not fully capture SP’s broader role in depression. These limitations warrant cautious interpretation and highlight the need for larger, more diverse studies to validate the findings and better understand SP’s involvement in major depressive disorder [47]. Additionally, Substance P is anatomically localized in brain regions associated with depression, such as the raphe nucleus, locus coeruleus, and nucleus accumbens [6,7,8]. Substance P influences neurotransmitter activity in these regions, contributing to depressive symptoms [61]. Further research involved recording activity from locus coeruleus neurons in a slice preparation, allowing precise drug application. Substance P was found to stimulate these neurons in a dose-dependent manner, primarily through its C-terminal region. Results suggest that the receptors in the locus coeruleus mainly respond to Substance P-P type. Interestingly, a synthetic analog of Substance P did not block Substance P‘s effects on these neurons [61].

Furthermore, neurokinin 1 receptors, which bond Substance P at high affinity, are distributed in regions of the brain involved in emotional development and regulation, specifically the hippocampus, hypothalamus, amygdala, and prefrontal cortex [62,63]. One study investigates the role of neurokinin 3 receptors (NK3-R) in learning, memory, and emotionality. Despite limited understanding, recent research suggests NK3-R involvement in various cognitive and emotional processes. Aging often leads to cognitive decline akin to mild cognitive impairment or Alzheimer’s disease, with aged rats serving as models for such conditions [62]. The study examines how the NK3-R agonist senktide affects learning, memory, emotionality, and cholinergic neurotransmission in aged rats. Tests include an episodic-like memory task, as well as open-field, and forced-swimming tests to assess memory, anxiety, and depression-like behaviors, respectively. Cholinergic neurotransmission changes in brain regions related to emotion and memory are also measured using in vivo microdialysis with HPLC-ECD after senktide administration [62].

Studies have also linked decreased white matter integrity in various brain regions with elevated Substance P levels, suggesting a role in structural brain changes associated with MDD [64]. The study that examined this possible relationship found a positive correlation between elevated levels of Substance P and decreased integrity of white matter within multiple regions of the brain, specifically in the prefrontal cortex, anterior cingulate cortex, and superior/inferior longitudinal fasciculus [57]. As many of these regions are crucial in higher cortical functions and emotional regulation, the study concluded that degeneration of these regions predisposed patients to major depressive disorder symptoms [64]. The study also suggests that increases in SP efflux, observed in response to stressors, serve as a direct indicator of SP neurotransmission in brain regions [64]. This implies that SP efflux could be considered a marker for SP activity during stress. Additionally, elevated levels of serum and cerebrospinal fluid SP in individuals with MDD imply that SP levels might serve as markers for this disorder [64]. The study’s sample size, though adequate for certain analyses, may limit generalizability. Recruitment from a specific psychiatric clinic and community ads could introduce selection bias. Exclusion criteria, like omitting those with comorbid psychiatric conditions, may limit representativeness. Reliance on self-reported data for variables such as handedness and ethnicity could introduce reporting bias. The cross-sectional design hinders establishing causal relationships. Despite efforts to standardize SP measurement, variations in assay performance may introduce measurement bias. While offering valuable insights, these limitations and biases should be acknowledged when interpreting findings [64].

Furthermore, neurokinin receptor antagonists and antidepressants have shown some efficacy in MDD treatment, underscoring the involvement of Substance P in depression [65,66,67]. Studies have also demonstrated that NK1 knockout mice models show similar results to patients with chronic treatment for depression [14]. NK1 receptor antagonists also appeared to have an effect on the amygdala, a key area of emotional regulation and seemingly assisting in the amelioration of depressive symptoms [65]. In addition, the study which measured CSF and serum levels of Substance P also measured the levels following antidepressant treatment [47]. They found that at the two and four-week marks following administration of the antidepressant, the levels of Substance P were significantly decreased compared to those before antidepressant administration. These findings led to the conclusion that Substance P played a role in the development of depressive symptoms [47].

**Table 2:** A brief summary of Relevant NK antagonist clinical trials [68,69,70,71].

Trial	Subjects	Methods	Measure of Outcome	Results
Orvepitant in Adult Post Traumatic Stress Disorder [68].	Male and female outpatients between the ages of 18 and 64 with a diagnosis of non-combative PTSD	Double-blind, placebo-controlled, fixed-dose administration; Placebo vs. Orvepitant 60 mg/day	Change in baseline in the Clinician-Administered PTSD Symptom Severity Scale from day 1 (predose) to week 12	A decrease in the mean difference of change on the PTSD Symptom Scale was seen in patients who were given Orvepitant in comparison to the placebo.
Substance P Antagonist in the Treatment of Posttraumatic Stress Disorder [69].	Male and female patients between the ages of 18 and 65 with a diagnosis of PTSD	Double-blind, placebo-controlled; placebo vs. vofopitant	Change in baseline in the Clinician-Administered PTSD Symptom Severity Scale from day 1 (predose) to week 8	A decrease in the mean difference of change on the PTSD Symptom Scale was seen in patients who were given vofopitant in comparison to the placebo.
Effect of LY686017 [70].	Male and female patients between 21 and 65 who meet the criteria for alcohol dependence and who have an elevated score on the general test of anxiety	Double-blind, placebo-controlled; placebo vs. 50 mg tradipitant	Change in baseline in the Alcohol Urges Questionnaire at day 1 to week 8 and biweekly assessment using the Comprehensive Psychiatric Rating Scale	A decrease in the mean difference of change in the Alcohol Urges Questionnaire in patients given tradipitant; a decrease in the mean difference of change in the Comprehensive Psychiatric Rating Scale in patients given tradipitant
A Randomized, Double-Blind, Parallel-Group, Placebo-Controlled, Fixed Dose Study Evaluating the Efficacy and Safety of Orvepitant in Subjects with MDD [71].	Male and female outpatients between 18 and 64 who have a primary diagnosis of MDD	Randomized, double-blind, parallel-group, placebo-controlled, fixed-dose; placebo vs. orvepitant	Change in baseline in the Hamilton Depression Rating Scale from day 1 to week 6	A decrease in the mean difference of change on the Depression Rating Scale in those patients given orvepitant

While NK antagonists demonstrate a possible avenue for treatment of conditions involving Substance P, there are some considerations, concerns, and limitations to their use currently. One limitation and concern is the lack of a significant number of clinical trials that have been performed to test NK antagonists against the current treatment models for both pain and psychiatric conditions. Furthermore, in general, there is also a shortage of studies and clinical trials concerning Substance P and NK antagonists. As mentioned above, Table 2 exhibits some of the relevant clinical trials that have been performed in recent years concerning the use of NK antagonists in various conditions [68,69,70,71]. These trials have demonstrated a marked decrease in patient-reported symptoms associated with conditions such as MDD, anxiety, and PTSD when patients were given an NK antagonist compared to those who received a placebo drug [68,69,70,71]. Given Substance P’s relationship to neurokinin receptors, the success of NK antagonist usage in these clinical trials points to possible effectiveness in other Substance P-associated conditions [68,69,70,71]. However, there are limitations to this theory as the table illuminates the paucity of trials focused on Substance P and its associated conditions discussed in this manuscript. This lack of significant clinical trials concerning Substance P provides the impetus for future research into their efficacy and use. Instead, much of the current research on the application of NK1 antagonists has centered on their role as antiemetics in chemotherapy and post-operative nausea patients [72].

**Figure 3:**
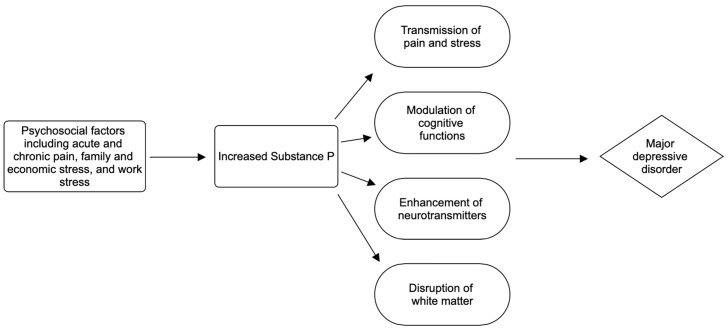
Model for potential mechanism of Substance P’s effects in the development of major depressive disorder [47,55,56,57,58,59,60,61,62,63,64].

Concurrently, consideration for the possible use of NK antagonists in treating conditions associated with Substance P should also consider the side-effect profile. With the use of NK antagonists, there have been reports of several serious side effects which could affect their widespread use, including Stevens–Johnson syndrome, neutropenia, angioedema, and QT prolongation [72]. Furthermore, NK antagonists also have milder side effects, including insomnia, loss of appetite, and hair loss [72]. Further research is necessary not only to discover the prevalence of these side effects but also to further solidify the relationship between Substance P and NK antagonists to open up avenues for potential treatment and prevention. A model for the potential mechanism of Substance P’s effect in the development of MDD is shown in Figure 3. 

## 7. Anxiety Disorders

Anxiety disorders are prevalent in the Western world, affecting approximately 19% of individuals in the United States [73]. Like MDD, anxiety disorders are closely linked with stress and pain [74]. Anxiety disorders involve an imbalance of neurotransmitters, including norepinephrine, serotonin, dopamine, and GABA [75]. These imbalances, coupled with abnormal activity in brain regions like the amygdala and prefrontal cortex, contribute to anxiety symptoms [76]. Stressors trigger an increase in endogenous Substance P levels in the CNS, particularly in regions like the amygdala, exacerbating anxiety symptoms [15]. Consequently, research and additional studies have explored the role of Substance P in anxiety disorder development and modulation. Studies dealing with psychiatric disorders face certain methodological challenges and biases that warrant consideration. Age and gender composition of the cohort may affect the correlations observed. Geriatric studies often focus on subclinical depression, limiting generalization. However, unexpected positive associations between subclinical symptoms and gray matter volume may challenge traditional assumptions. Gender effects and nonlinear relationships between brain structure and symptoms are also important. Overall, caution is needed in interpreting neuroimaging data in mood disorders [76].

A study investigated how emotional stress affects Substance P (SP) release in different amygdala regions in rats [15]. They found that immobilization stress led to a significant and lasting increase in SP release in the medial amygdala (MeA), while exposure to a mild stressor transiently boosted SP release in the MeA [15]. Furthermore, this study also showed that blocking the neurokinin-1 receptor in the MeA prevented stress-induced anxiety-like behavior, suggesting the importance of SP release in this brain area for anxiety responses. These findings highlight the potential of SP antagonists in treating stress-related disorders [15]. Similar studies demonstrated that microinjections of Substance P into critical regions of the brain produce anxiogenic-like behaviors in animal models [15]. Further studies demonstrated that Substance P also activates the sympathetic nervous system and the hypothalamic–pituitary–adrenal (HPA) axis [10]. Stimulation of both of these areas increases and enhances the effects of neurotransmitters such as norepinephrine and cortisol, leading to anxiogenic effects within an individual [10]. Similarly to MDD, neurokinin receptor antagonists exhibit anxiolytic effects, suggesting a role for Substance P in anxiety disorders [10,77]. 

## 8. Post-Traumatic Stress Disorder

PTSD is a debilitating psychiatric condition that often arises following exposure to life-threatening situations or severe trauma [78]. Some epidemiological investigations have unveiled elevated rates of physical comorbidities associated with immune dysregulation among PTSD sufferers, encompassing conditions such as metabolic syndrome, cardiovascular disease, and autoimmune disorders [78]. A study has shown elevated concentrations of interleukin-1β, interleukin-6, tumor necrosis factor-α, and C-reactive protein in PTSD patients. These markers are indicative of an activated immune and inflammatory response in individuals experiencing PTSD-related symptoms [79]. PTSD also often occurs following a stroke, yet the specifics of its progression and the underlying neurochemical factors and brain circuits remain unclear [80]. Its diagnosis relies on identifying classic symptoms classified into four clusters: re-experiencing, avoidance, negative cognitions and mood, and arousal [81]. Screening tools like the Breslau Short Screening Scale and the PTSD Checklist aid in the accurate detection of PTSD [82,83]. The use of brief screening instruments in low- and middle-income countries (LMICs) has the potential to raise awareness and research opportunities for various at-risk populations [84].

Additionally, a recent study investigated the intricate relationship between childhood trauma, parental bonding, psychopathology, impulsivity, resilience, and neuropeptide NPY and SP levels in individuals with a history of opioid use. Results highlighted associations between antisocial features, depression, impulsivity, emotional neglect, resilience, and severity of drug-related problems. Particularly noteworthy was the correlation between SP levels and antisocial personality traits. These findings suggest the potential utility of serum NPY and SP levels as biomarkers to predict behavioral adjustment and the severity of drug-related issues in opioid users. This study allows one to see the potential for integrating biological vulnerabilities with childhood risk factors [85].

Recent research has identified imbalances in serotonin and Substance P levels in individuals with PTSD [86]. Elevated Substance P levels within the CNS, particularly in response to traumatic triggers, suggest a potential avenue for exploring Substance P antagonists in PTSD treatment [46]. This study’s small sample size, reliance on medication-free individuals, and within-subject design may limit generalizability and introduce confounding factors. Additionally, using CSF Substance P concentrations as a sole biomarker and a videotape stimulus for trauma induction could oversimplify complex conditions and experiences, potentially affecting the validity of the findings [46]. Another study has further implicated Substance P as a role player in conditions associated with PTSD, such as traumatic brain injuries (TBIs) [87]. This recent study demonstrated that the use of NK1 antagonists significantly reduced the cognitive and motor complications and deficits of TBIs, including the development of psychiatric conditions [87]. Given that one of the main actors on the NK1 receptor is Substance P, a reasonable conclusion can be drawn that Substance P plays a role in the development of psychiatric conditions, including PTS. This conclusion may be especially true in predisposed patients, such as those who have suffered from traumatic brain injuries.

A recent meta-analysis explored the relationship between post-traumatic stress disorder (PTSD) and irritable bowel syndrome (IBS) [88]. The meta-analysis, highlighted the interplay of psychological factors with chronic pain (PTSD) and irritable bowel syndrome (IBS), providing insight into the interplay of psychological factors with chronic pain [88]. This comprehensive analysis, which examined eight studies involving a total of 648,375 subjects, uncovered a significant association between PTSD and IBS. The findings, presenting a pooled odds ratio of 2.80 (with a 95% confidence interval ranging from 2.06 to 3.54), highlight the significance of recognizing the psychosocial dimensions of chronic pain ailments [46]. They highlight the imperative for a holistic approach to managing pain, one that acknowledges the impact of psychological factors. Furthermore, these results suggest that individuals grappling with PTSD may face an elevated risk of developing concurrent chronic pain disorders. This emphasizes the need for further exploration into multifaceted therapeutic interventions that address both the physical and psychological aspects of pain management [88].

## 9. Conclusions

This review delineates the myriad ways in which Substance P impacts disease development, highlighting its significant role in pain and psychiatric conditions. Substance P exerts its effects through multiple mechanisms, including enhancing transmission, increasing receptive field size, and decreasing neuronal thresholds. These actions contribute to the transmission and development of acute and chronic pain. While many diseases are associated with acute and chronic pain, this review focuses on Substance P’s role in psychiatric conditions, specifically major depressive disorder (MDD), anxiety, and post-traumatic stress disorder (PTSD). Elevated levels of Substance P are implicated in these psychiatric diseases, and microinjections of Substance P have been found to reproduce symptoms akin to these conditions. Moreover, a positive correlation between elevated Substance P levels and decreased white matter volume has been observed in patients with psychiatric conditions such as MDD. The role of Substance P in psychiatric diseases is further supported by studies showing that neurokinin antagonists can ameliorate the symptoms of these disorders. Given the demonstrated effects of Substance P and the therapeutic potential of neurokinin antagonists, further clinical trials and studies are warranted. These could provide alternative treatment avenues for patients suffering from psychiatric conditions like MDD and anxiety. Additionally, the prevalence of increased Substance P in patients experiencing both pain and psychiatric conditions suggests its potential as a biomarker for psychiatric disorders. This could offer a valuable diagnostic tool for guiding practitioners in the prevention and treatment of these disorders. In summary, this review has illuminated potential research avenues that could enhance patient care. Future studies exploring the mechanisms of Substance P, its role as a biomarker, and the therapeutic potential of neurokinin antagonists could significantly impact the treatment and management of pain and psychiatric disorders.

## Figures and Tables

**Table 1:** Exploring the role of Substance P and associated receptors in animal models [12,14,15,16].

Study	Animal Models	Experimental Conditions	Measurement of Outcome	Conclusions
Bilkei-Gorzo et al. (2002) [14].	*Tac1* mutant mice and wild-type mice	Exposure to stress with a forced-swimming test, tail-suspension test, bulbectomy, social interaction test, open-field test	Video recordings of animal behavior with an observer measuring immobility, hyperactivity, distance traveled in an open field, and social interaction with other animals	Mice that did not have the tac1 gene that encodes for Substance P displayed less fear and anxiety and were also more active in depression-related paradigms
Ebner et al. (2004) [15].	Adult male *Sprague-Dawley* rats	Microinjections of Substance P and NK antagonists as well as immobilization with stress exposure	Measurement of Substance P concentrations by in vivo micro push–pull superfusion and microdialysis; behavior was measured by activity in different arms of a maze	Significantly increased Substance P release in rats exposed to stress in comparison to rats that were not, as well as NK1 antagonist application leading to decreased stress-induced anxiolytic effects
Bradesi et al. (2009) [12].	Male *Wister* rats	Application of water and sham stress	Western blotting with antibodies for NK1 receptors	Upregulation of NK1 receptors and hyperalgesia in mice exposed to stress
Drew et al. (2005) [16].	Adult male and female *Sprague-Dawley* rats	Application of drug solutions containing Substance P on neurons within the PAG and RVM	Measurement of the IPSCs and EPSCs of neurons	Increased Substance P levels in the PAG led to modulation of descending pain pathways and analgesia
